# Anti-Amyloid Therapy, AD, and ARIA: Untangling the Role of CAA

**DOI:** 10.3390/jcm12216792

**Published:** 2023-10-27

**Authors:** Mo-Kyung Sin, Edward Zamrini, Ali Ahmed, Kwangsik Nho, Ihab Hajjar

**Affiliations:** 1 College of Nursing, Seattle University, Seattle, WA 98122, USA; 2 Irvine Clinical Research, Irvine, CA 92614, USA; ezamrini@irvineclinical.com; 3 VA Medical Center, Washington, DC 20242, USA; ali.ahmed@va.gov; 4 School of Medicine, Indianna University, Indianapolis, IN 46202, USA; knho@iupui.edu; 5 School of Medicine, University of Texas Southwestern, Dallas, TX 75390, USA; ihab.hajjar@utsouthwestern.edu

**Keywords:** amyloid β, Alzheimer’s disease, anti-amyloid therapy, cerebral amyloid angiopathy, CSF, plasma

## Abstract

Anti-amyloid therapies (AATs), such as anti-amyloid monoclonal antibodies, are emerging treatments for people with early Alzheimer’s disease (AD). AATs target amyloid β plaques in the brain. Amyloid-related imaging abnormalities (ARIA), abnormal signals seen on magnetic resonance imaging (MRI) of the brain in patients with AD, may occur spontaneously but occur more frequently as side effects of AATs. Cerebral amyloid angiopathy (CAA) is a major risk factor for ARIA. Amyloid β plays a key role in the pathogenesis of AD and of CAA. Amyloid β accumulation in the brain parenchyma as plaques is a pathological hallmark of AD, whereas amyloid β accumulation in cerebral vessels leads to CAA. A better understanding of the pathophysiology of ARIA is necessary for early detection of those at highest risk. This could lead to improved risk stratification and the ultimate reduction of symptomatic ARIA. Histopathological confirmation of CAA by brain biopsy or autopsy is the gold standard but is not clinically feasible. MRI is an available in vivo tool for detecting CAA. Cerebrospinal fluid amyloid β level testing and amyloid PET imaging are available but do not offer specificity for CAA vs amyloid plaques in AD. Thus, developing and testing biomarkers as reliable and sensitive screening tools for the presence and severity of CAA is a priority to minimize ARIA complications.

## 1. Introduction

Alzheimer’s disease (AD) is the leading cause of dementia in older adults. As the U.S. population ages, the incidence and prevalence of AD will increase, contributing to disability among older adults [1]. Identifying ways to slow down cognitive decline and prolonging the duration of early-stage AD is desperately needed to promote quality of life in older adults. Anti-amyloid therapies (AATs) are emerging as treatment options for early AD, targeting amyloid β in the brain. Amyloid β plays a key role in the pathogenesis of AD and cerebral amyloid angiopathy (CAA). Amyloid-related imaging abnormalities (ARIA), abnormal signals seen on MRI of the brain in patients with AD, are major side effects of anti-amyloid therapies. CAA is a major risk factor for ARIA [2]. As the use of AAT treatments grows, a better understanding of the pathophysiology of ARIA and identification of those at highest risk is necessary to optimize treatment outcomes. This review provides an overview of AATs, risk factors, and potential CAA screening tools for health care providers in the field of AD treatment and management.

## 2. Amyloid β Plays a Key Role in the Pathogenesis of AD and CAA

AD is the 7th leading cause of death in the U.S. and the most common cause of dementia among older adults [3], affecting approximately 6.7 million Americans aged 65 and older in 2023 [1]. Accumulation of amyloid β in the brain plays a key role in the pathogenesis of AD, synaptic dysfunction, neurodegeneration, and finally clinical symptoms [4,5,6]. Thus, emerging AD medications primarily target the halting of amyloid β formation or its removal.

Amyloid β is made up of several species of 39–43-residue peptides (including amyloid β1-40 and amyloid β1-42) that are produced from amyloid precursor protein (APP) [7,8]. Soluble amyloid β is produced throughout life but some fragments become pathologically insoluble and aggregate into fibrils, which combine further to form neuritic plaques and vascular deposits of amyloid β [9]. Amyloid β accumulation in the brain parenchyma as plaques is a pathological hallmark of AD [8,10]. Amyloid β accumulation in cerebral vessels, primarily in the medium-sized arteries and arterioles in the leptomeningeal and cortical regions, leads to CAA [9,11,12,13,14,15].

CAA can occur in certain familial syndromes or can occur spontaneously. Familial CAA is caused by mutations in the APP gene [16]. Although the exact cause is not known for sporadic CAA, old age and ApoE e4 have shown associations [16]. Amyloid β1-42 is thought to be the foundation for both parenchymal plaques and CAA formation. Higher cerebral amyloid β1-40 levels and amyloid β1-40/amyloid β1-42 ratios are more likely related to CAA formation, while higher amyloid β1-42 levels are more likely related to parenchymal plaques [13,14,15]. CAA contains both amyloid β40 and amyloid β42 in contrast to parenchymal deposition. However, amyloid β40 is the major isoform in CAA with increased accumulation as its severity progresses. In a previous study, CSF amyloid β40 and amyloid β42 were analyzed in 72 AD patients and 58 controls to examine whether they can serve as molecular biomarkers for CAA. They found decreased CSF amyloid β40 and amyloid β42 in CAA patients relative to controls and AD patients [17]. CSF analysis was helpful in differentiating CAA from controls but had a limitation in differentiating CAA from AD [17]. Table 1 below is an overview of clinical, imaging, biomarker, and pathological differences between AD and CAA [16,18,19].

## 3. Anti-Amyloid Therapies Are Emerging as Treatments for AD

AATs, humanized second-generation monoclonal antibodies, are emerging as treatments for individuals with mild cognitive impairment (MCI) and mild dementia due to AD [20]. Anti-amyloid therapy targets insoluble and fibrillar amyloid β in the central nervous system (CNS) and favorably alters putative downstream biomarkers of CNS tau/tangle pathology, and thus has potential disease-modifying benefits [20]. FDA-approved anti-amyloid drugs include lecanemab and aducanumab [20]. Lecanemab, the most recently approved medication, is administered as weight-based intravenous infusions every 2 weeks over 18 months [21]. Most individuals on lecanemab displayed amyloid β-negative status on PET scans by 12 months of therapy [22]. In addition, those receiving lecanemab showed, on average, an approximately 25% slowing in cognitive decline over 18 months [23], equivalent to a 4–5-month delay in disease-related progression, compared to those receiving a placebo [21,23,24]. However, AATs are not cures for AD and there is no evidence that these drugs stop progression of cognitive impairment. Also, these medications are only for those with MCI and mild dementia due to AD who show evidence of a buildup of amyloid β as plaques in the brain, and are excluded treatments for those with moderate or severe dementia and dementia from other causes.

## 4. Amyloid-Related Imaging Abnormalities (ARIA) Are Significant Side Effects of AATs

Amyloid-related imaging abnormalities (ARIA), abnormal signals seen on MRI of the brain in patients with AD, may occur spontaneously [25,26,27], but occur more frequently in persons receiving AATs [21,28,29]. ARIA is classified into these 2 subtypes: ARIA-E [edema and/or effusion best detected using T2 fluid-attenuated inversion recovery (FLAIR) MRI] and ARIA-H (microhemorrhages, macrohemorrhages, and/or superficial siderosis best detected on susceptibility weighted imaging) [20,29]. Spontaneous ARIA may occur because of CAA and CAA-related vessel inflammation (also known as amyloid β-related angiitis) [30]. CAA-related vessel inflammation also occurs in a minority of people receiving anti-amyloid therapy by activated microglia, T cells, and amyloid β-containing multinucleated large cells surrounding CAA-positive vessel walls [30,31]. Impaired vascular wall integrity from increased severity of CAA leads to inflamed and weakened vessel walls, resulting in leakage of proteinaceous fluid and blood (ARIA-E or ARIA-H) [30]. Associated symptoms can range from benign to severe. Clinical symptoms of ARIA-E/H include the following: headache, changes in mental status, confusion, visual disturbances, vomiting, nausea, tremor, gait disturbances, and even death [20,21,29,32]. Symptomatic ARIA was <3% within the first 3 months for 10 mg/kg monthly and biweekly dosing regimens in a phase 2 lecanemab trial [28,29]. The incidence of imaging abnormalities with edema or effusions (ARIA-E) was 12.6% (vs. 1.7% with placebo) and cerebral microhemorrhages, cerebral macrohemorrhages, or superficial siderosis (ARIA-H) was 17.3% (vs. 9.05% with placebo) in a phase 3 lecanemab trial [21]. The severity of ARIA-E is graded based on the number and size of edematous regions on MRI, whereas the severity of ARIA-H is graded based on the number of microhemorrhages and superficial siderosis (Table 2) [30]. Table 2 below is the MRI rating scale for ARIA-E and ARIA-H [31]. 

Although the underlying mechanisms of ARIA have not been identified, possible causes may be a combination of increased permeability from increased amyloid β clearance and associated saturation of perivascular drainage [33,34], direct antibody interaction with deposited amyloid β, and weakened vascular walls. Rapid movement of amyloid into the cerebral vessel walls can cause increased vascular friability and permeability [35]. The current ARIA monitoring protocol for anti-amyloid therapy includes MRI within 1 year before medication initiation and before the 5th, 7th, and 12th dose infusions [36]. If a patient develops symptomatic ARIA E/H, monthly MRI is suggested after suspending the medication until resolution of ARIA-E and stabilization of ARIA-H [36]. When symptoms of ARIA-E/H resolve, therapy can resume after acquiring patient consent [36]. The only treatment currently available for symptomatic ARIA management is to reduce the dose, to discontinue treatment temporarily or permanently, and the potential use of steroids for severe ARIA-E. As AATs increasingly become treatment options, a better understanding of the pathophysiology of ARIA is necessary for early detection of those at highest risk and for the proper management of therapeutic regimens for those experiencing symptomatic ARIA. In addition, identifying those at highest risk will provide clinicians with an idea for stratified monitoring and, potentially, schedule and dosage adjustments.

## 5. CAA Is a Major Risk Factor for ARIA

Risk factors for ARIA include CAA, presence of one or more microhemorrhages/superficial siderosis at baseline, a higher baseline amyloid load, initial treatment period (e.g., most ARIA-E occurred within the first 3 months of treatment), type of antibody used, higher dosage, and ApoE e4 genotype (ApoE e4 homozygotes having the highest risk) [2,20,37,38,39,40,41]. ARIA is potentially driven by coexisting CAA [2]. Among the risk factors, CAA is the major risk factor for ARIA because CAA is highly prevalent in AD, and both are driven by impaired amyloid β clearance [2]. In addition, the imaging and pathological manifestations of ARIA are similar to CAA, supporting the idea that both share a common pathway [2].

Microbleeds and ApoE e4 presence at baseline indicate possible ARIA and CAA because microbleeds suggest the presence of amyloid β in vessel walls and ApoE e4 suggests a high amyloid β burden [42,43]. CAA-related vascular or perivascular inflammation is a spontaneously occurring condition with clinical and neuroimaging features similar to anti-amyloid related ARIA-E and commonly presents with symptoms [44,45], while anti-amyloid related ARIA-E is often asymptomatic [35]. Both have a significant association with ApoE e4 [35]. Thus, early identification and distinction between plaque and vascular amyloid could lead to strategies to reduce occurrence of ARIA in patients receiving anti-amyloid treatment. In addition, standardized protocols to facilitate uniform reporting of CAA in neuropathology and imaging studies may be useful to increase clarity and promote greater understanding of the condition.

**Figure 1:**
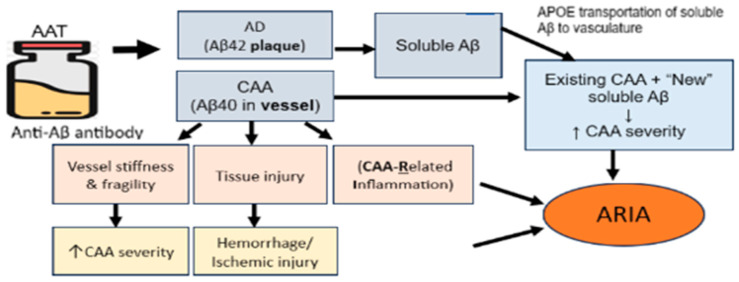
AATS, CAA, and ARIA Mechanism.

In the EMERGE and ENGAGE phase 3 randomized clinical trials of aducanumab, the incidence of ARIA-E was highest in the aducanumab 10 mg/kg group (362/1029, 35.2%) compared with the 6 mg/kg group (83/392, 21.2%), 3 mg/kg group (223/756. 29.5%), and placebo group (29/1076, 2.7%) [32]. ARIA has been observed most prominently in trials of antibodies that target amyloid β N-terminal compared to those that target mid-peptide and C-terminal regions of amyloid β [42,46,47,48]. For example, several clinical trials have reported an ARIA-E incidence of up to 55% in ApoE e4 (homozygous) carriers receiving the N-terminus amyloid β targeted medications [32,49,50] compared to treatments targeting the mid-domain or C-terminus of amyloid β [51,52]. The N-terminus is associated with soluble forms of Aβ, which can be more readily mobilized from brain tissue to the bloodstream, potentially leading to vascular or perivascular amyloid β accumulation and edema [46,47,48]. Despite the high incidence of ARIA, the N-terminal region targeted anti-amyloid medications have been found to be more effective in reducing amyloid β because those have the potential to clear more toxic forms of amyloid β. CAA is an important condition needing attention with the introduction of AATs for the treatment of AD. Amyloid deposition in vessel walls may lead to loss of vascular integrity and reduced perivascular clearance [31]. When AATs are initiated, antibody-mediated breakdown of amyloid plaque and mobilization of both vascular and parenchymal amyloid β increase the load of perivascular drainage. This overloaded perivascular pathway transiently increases amyloid deposition in the vessel walls [53]. At the same time, antibody-mediated inflammation and amyloid breakdown also occurs in the vessel wall, leading to further loss of vascular integrity and blood–brain barrier breakdown. As a result, proteinaceous fluid and/or red blood cells leak into the parenchyma and/or leptomeningeal space, resulting in edema/effusion (ARIA-E) or microhemorrhages/superficial siderosis (ARIA-H) [31]. Certain clinical and radiological features previously considered to represent AD, may in fact be manifestations of CAA; thus, future treatment of AD may be less efficacious in relieving cognitive impairment if the impact of CAA is not considered. Figure 1 below explains the underlying mechanisms of AATs, CAA, and ARIA.

## 6. CAA Is an Independent Risk Factor for Cognitive Impairment

CAA is distinct from age-related cerebral small vessel disease, which independently contributes to cognitive decline [2]. CAA was present in about 50–90% of people with AD in neuropathologic samples [54,55,56,57] and about 30% of older adults without AD or other neuropathological abnormalities [58]. In a study based on the Adult Changes in Thought autopsy cohort, CAA was present in 38% (*n* = 322) of 848 participants with and without dementia [53% (173/322) in dementia cases] [57]. In a study from the two longitudinal clinical-pathologic studies of aging, the Rush Memory and Aging Project and the Religious Orders Study, CAA pathology was greater among persons with AD dementia (Wilcoxon test, *z* = 7.912, *p* < 0.0001) compared to persons without dementia [59]. The high prevalence of CAA in AD places many patients at risk for ARIA-associated morbidity.

The presence of CAA is related to hemorrhages ranging from micro-to-macro, potentially resulting in further cognitive impairment and even death [60,61,62,63]. Advanced CAA can lead to vessel fragility and rupture, resulting in intracerebral hemorrhage in lobar regions, cortical superficial siderosis, and white matter hyperintensities (WMH) [64,65,66]. Cerebral microhemorrhage, cortical superficial siderosis, and a history of intracranial hemorrhage are well-established surrogate markers of CAA severity.^28^ Compared to the frontal predominance of WMH in normal elderly [67,68] occipital-predominant white matter lesions are frequent in people with CAA [69,70,71,72].

## 7. Age and ApoE e4 Are Important Risk Factors for CAA

Advancing age is the strongest risk factor for CAA [73]. CAA is occasionally diagnosed in people in their 50s or 60s but is much more common in people in their 70s and 80s [1]. The ApoE e4 allele has been found to be an important risk factor for AD as well as for CAA formation. It is believed that ApoE e4 promotes vascular amyloid accumulation and thus increases the odds for moderate or severe CAA [74,75,76,77]. Data from the National Alzheimer’s Coordinating Center reported that people with ApoE e4 were more likely to have CAA (X^2^(3) = 150.6, *p* < 0.001) [62]. Among 371 autopsy samples, 81% of ApoE e4/e4 carriers had severe CAA in the parenchyma and 95% of ApoE e4 homozygotes carriers had severe CAA in the meninges of the occipital lobe [74]. Autopsy data from the Religious Orders Study and the Rush Memory and Aging Project reported that ApoE e4 carriers had tripled odds (OR = 3.55, 95% CI = 2.73−4.63, *p* < 0.001) of having more severe meningeal/parenchymal CAA than the ApoE e3/e3 reference group [75]. In that study, 80% of the brains with moderate to severe CAA met pathologic AD criteria [75]. CAA can be present with or without capillary involvement. ApoE e4 carriers are more likely to have capillary CAA (capillary amyloid β-deposition) which is strongly associated with AD cases [75,78].

## 8. Transient Focal Neurological Episodes Are the Earliest Clinical Symptoms in CAA

CAA-related transient focal neurological episodes (TFNEs) are brief (typically lasting 10–30 min) and recurrent disturbances in motor, somatosensory, visual, or language functions (e.g., paralysis, weakness, loss of muscle control, increase or loss of muscle tone, involuntary movement such as tremor, or sensory changes such as abnormal sensations, numbness, or decreases in sensation) often spreading from one body part to another [71]. TFNEs may mimic transient neurologic symptoms such as transient ischemic attack, migraine with aura, focal seizure, structural lesions (e.g., tumor, vascular malformation, subdural hematoma), metabolic abnormalities (e.g., hypoglycemia, hyponatremia), syncope or presyncope, and functional neurologic disorder [79]. TFNEs are increasingly recognized as the earliest clinical symptoms in people with CAA [66,69,70,71,79]. Other major complications of CAA include intracranial hemorrhage, transient neurological symptoms (e.g., changes in mental status, headaches, seizures, focal neurological signs or hallucinations), cortical superficial siderosis, and CAA-related inflammation [16,80].

## 9. A Sensitive and Reliable Diagnostic Biomarker for CAA Is Needed

Careful prescreening for CAA in anti-amyloid therapy candidates is necessary to minimize ARIA and promote best treatment outcomes. Not all cases of CAA can be identified presymptomatically. Identifying specific and sensitive diagnostic markers for CAA that could be used before initiating anti-amyloid therapy is an urgent priority. In addition, evaluating CAA severity scores against meaningful clinical outcomes will be essential to identify whether this adds to the predictive value of individual markers or has a practical application in clinical practice or trials [61].

The gold standard for CAA diagnosis is histopathological confirmation by brain biopsy, which is not clinically feasible [81]. Conventional computed tomography (CT) and MRI are the most reliable in vivo tools in identifying CAA according to the modified Boston criteria [82]; however, they detect only the secondary consequences of CAA such as microbleeding and WMH, not the vascular amyloid itself nor the severity of CAA [17]. In addition, current imaging markers lack specificity because those markers may be partially caused by arteriosclerotic small vessel disease [83]. In the absence of direct neuropathological examination, the most commonly used criteria for CAA diagnosis are the modified Boston criteria (probable CAA being the most commonly used diagnostic category) based on clinical and MRI data [80]. The most recently updated version (2.0) of the Boston criteria for the diagnosis of probable CAA in patients aged 50 years and older include the following clinical and MRI data: (1) presentation with spontaneous hemorrhage, TFNEs, or cognitive impairment or dementia, (2) at least two of the following strictly lobar hemorrhagic lesions on T2-weighted MRI, in any combination: intracranial hemorrhage, cerebral microbleeds, or foci of cortical superficial siderosis or convexity subarachnoid hemorrhage, OR (1) one lobar hemorrhagic lesion plus one white matter feature, (2) absence of any deep hemorrhagic lesions, (3) absence of other cause of hemorrhagic lesions, and 4) hemorrhagic lesion in cerebellum not counted as either lobar or deep hemorrhagic lesion [81]. Amyloid PET is unable to distinguish between vascular and parenchymal amyloid β [61].

Since CAA results from the impaired clearance of amyloid β from interstitial cerebral fluid, CSF should contain biomarkers reflecting this process [61]. CAA-affected cerebral vessels contain significant amounts of amyloid β40; thus, reduced levels of amyloid β40 in CSF and plasma is a surrogate marker for CAA, whereas reduced levels of amyloid β42 in CSF and plasma is a surrogate for AD [84,85]. Decreasing CSF amyloid β40 concentrations were associated with higher lobar microbleed count, increasing WMH volume, and the presence of cortical superficial siderosis [86]. Another study found a relationship between increased cerebral amyloid burden (lower CSF Aβ42 levels) and WMH occurrence in specific posterior white matter regions [87,88]. In addition, the volume of white matter abnormalities began to increase prior to clinical symptom onset from AD [89]. WMH and cortical microinfarcts are ischemic manifestations of CAA, are the earliest makers of the hereditary form of CAA, and precede intracranial hemorrhage [90]. Several studies have tested CSF biomarkers such as amyloid β40 and amyloid β42 to identify early-stage CAA [19,83,91,92,93] and have reported conflicting results. All agreed that CSF amyloid ß40 and amyloid ß42 levels were decreased in people with CAA compared to both control and AD groups but inconclusive in the ability to distinguish CAA from AD.

Studies found that reduced levels of plasma amyloid β40 were associated with WMH in sporadic CAA, supporting circulating amyloid β as a potential indicator of cerebral microvascular damage [86,94]. A recent study conducted in rats found reduced CSF and plasma levels of amyloid β40 serving as a biomarker for early-stage CAA prior to the onset of cerebral microbleeds on MRI and histological staining. The levels of plasma and CSF amyloid β40 levels precipitously dropped at the early onset of CAA and continued to decrease with the progression of disease [84]. Plasma amyloid β has not been tested for CAA in humans. While the plasma test is a rapid, minimally invasive, and inexpensive method with high accuracy for AD, particularly when combined with age and ApoE e4 carrier status [95,96,97], studies are needed for CAA in humans.

## 10. Conclusions

CAA is an important condition needing attention following the introduction of AATs for the treatment of AD. Considering high prevalence of CAA among individuals with AD, taken together with the high risk of ARIA in patients with AD and CAA receiving AATs, careful screening for CAA among patients with AD may help minimize the side effect of AATs. Thus, developing and testing biomarkers as a reliable and sensitive screening tool to assess the presence and severity of CAA is a priority and is essential for optimal patient care and outcomes. The plasma β test is a rapid, minimally invasive, and inexpensive method and may serve as a potential diagnostic tool for CAA, based on the results of a recent rat experiment. Future studies are needed for the assessment and validation of plasma amyloid β and other strategies for screening CAA in patients with AD.

## Figures and Tables

**Table 1:** Overview of Differences between AD and CAA.

Overview	AD	CAA
Common clinical symptom	Memory disorders	Intracranial hemorrhage
Imaging finding	Hippocampal atrophy	Hemorrhage (macrobleed in very severe cases; multiple microbleeds in chronic, late-stage); cortical superficial siderosis
Biomarkers findings (CSF, plasma)	↓ Amyloid β42, ↑ amyloid β40, ↓ amyloid β42/40, ↑ total tau (t-tau), ↑ phosphorylated tau (p-tau^181^)	↓ Amyloid β40, ↑ amyloid β42, ↓ amyloid β40/42, ↓ total tau, ↓ phosphorylated tau 181 (p-tau^181^)
Neuropathological findings	Amyloid β accumulation in parenchyma as plaques	Amyloid β accumulation in the leptomeninges and small to medium-sized cerebral vessels

**Table 2:** MRI Rating Scale for ARIA-E and ARIA-H.

ARIA Type	Radiological Severity		
	Mild	Moderate	Severe
ARIA-E			
Size	<5 cm	5–10 cm	>10 cm
Location	Limited to a single site within sulcus or cortex/subcortical white matter	One or multiple brain locations	Significant involvement in the sulcus or subcortical white matter in one or more distinct sites
ARIA-H			
New incident of microhemorrhages	≤4	5–9	≥10
Focal areas of superficial siderosis	1	2	>2

## Data Availability

Not applicable.
